# New light on Smac mimetics and breast cancer

**DOI:** 10.1038/cddis.2016.81

**Published:** 2016-04-07

**Authors:** L Cornmark, C Larsson

**Affiliations:** 1 Department of Laboratory Medicine, Translational Cancer Research, Lund University, Medicon Village, Building 404:C3, Lund SE-22381, Sweden

The evasion of cell death is an important contribution to cancer development and may also confer resistance to chemo and radiotherapy.^1^ Therefore, reinstating a functional cell death machinery is one potential strategy for cancer therapy. One way to promote cell death induction is to use Smac mimetics (SMs),^2^ which are small molecules mimicking the proapoptotic protein Smac. Smac inhibits members of the inhibitor of apoptosis protein (IAP) family of which cellular IAP 1 (cIAP1) and 2 (cIAP2) are important for apoptotic signaling involving the death receptor TNF*α* receptor 1 (TNFR1). SMs, thereby, re-direct the TNFR1 from NF-*κ*B signaling towards apoptosis. This is achieved by downregulating, and thus inhibiting, cIAP1 and cIAP2 and their function as ubiquitinators of proteins recruited to the TNFR1. Without cIAP-mediated ubiquitination of proteins such as receptor interacting protein 1 (RIP1), the NF-*κ*B signaling cannot be conveyed. Instead, a complex containing RIP1 recruits caspase 8, activating the caspase cascade and apoptosis. In addition to redirecting the TNFR1, SM-mediated downregulation of cIAPs also leads to accumulation of NF-*κ*B inducing kinase (NIK) and the initiation of the noncanonical NF-*κ*B signaling pathway, which can result in induction of TNF*α* synthesis and possible stimulation of the TNFR1 in an autocrine manner. Whether SM treatment leads to upregulation of TNF*α* or not has been suggested to determine sensitivity to SMs.^3, 4, 5^

Breast cancer is the most common cancer for women worldwide. Screening protocols and advances in therapy have resulted in a 30% increase in 5-year survival between 1975 and 2005 to 90%.^6^ However, for basal-like breast cancers, which typically are estrogen- and progesterone receptor-negative as well as lack ERBB2 amplification, there is still no targeted treatment and they are more difficult to treat.^7, 8^ Therefore, strategies to treat this patient group are warranted. Some basal-like breast cancer cell lines, such as MDA-MB-231 are indeed sensitive to SMs suggesting that this pathway may be a potential target for therapy. However, most cell lines are resistant to SM as single treatment. Now, Cornmark *et al.*^9^ present a report demonstrating that basal-like breast cancer cell lines can be sensitized to SMs by activating protein kinase C (PKC) (Figure 1). They report that combining the PKC activator TPA with SM leads to cell death in three different basal-like breast cancer cell lines. The cell death can be blocked by using a TNF*α*-blocking antibody indicating the importance of TNF*α* in eliciting cell death. When investigating TNF*α* more closely, they found that PKC activity resulted in increased levels of TNF*α*, both at the mRNA and protein levels. The PKC-induced *de novo* production of TNF*α* was shown to be mediated by the canonical NF-*κ*B pathway^9^ (Figure 1).

It has previously been described that cancer cell lines sensitive to SM as a single agent are dependent on autocrine TNF*α* production. However, a majority of cancer cell lines are insensitive to SMs that seems to be due to an inability to induce TNF*α* expression.^3^ To investigate mechanisms explaining differences in Smac sensitivity, the response to SM in sensitive MDA-MB-231 and insensitive MDA-MB-468 breast cancer cell lines was compared. One striking difference was that the sensitive cell line had many more upregulated genes after SM treatment compared with the insensitive cell line where few genes were differentially expressed. However, both cell lines responded to SM with increased expression of the *BIRC3* gene that encodes cIAP2. Furthermore, both cell lines responded to SM in the same way by initiating noncanonical signaling as detected by p100 processing and p52 nuclear translocations.

The noncanonical NF-*κ*B signaling pathway has been suggested as the mediator of SM-induced TNF*α* production.^3, 4, 5, 10^ Nevertheless, in MDA-MB-468 cells, SM induces the noncanonical pathway and gene expression (*BIRC3* induction), but still no increase in TNF*α*. This raises the possibility that other pathways are critical for SM-mediated TNF*α* synthesis. Therefore, Cornmark *et al.*^9^ used a siRNA approach to target either NIK and the noncanonical NF-*κ*B pathway and/or IKK*β* and the canonical NF-*κ*B pathway. They found that only downregulation of both pathways resulted in a decrease in SM-mediated TNF*α* production that also resulted in a suppression of SM-induced cell death. However, although the absolute TNF*α* levels were suppressed, the relative induction still persisted as TNF*α* levels were markedly suppressed by the siRNAs also in unstimulated cells. This suggests that pathways other than NF-*κ*B may be necessary to obtain sufficient TNF*α* levels to induce cell death.

Thus, Cornmark *et al.*^9^ have shown that the SM-sensitive breast cancer cell line MDA-MB-231 needs either the canonical or the noncanonical pathway to mediate TNF*α* induction and cell death. Even if both pathways are suppressed, SM can still increase TNF*α* synthesis to some extent, suggesting that other mechanisms are also involved.

Taken together, these results further support previous studies^2^ that a simultaneous induction of TNF*α* may be one strategy to increase SM sensitivity. In basal-like breast cancer cells, this can be achieved by PKC activation that may open up for novel ways to target this group of breast cancers.

## Figures and Tables

**Figure 1:**
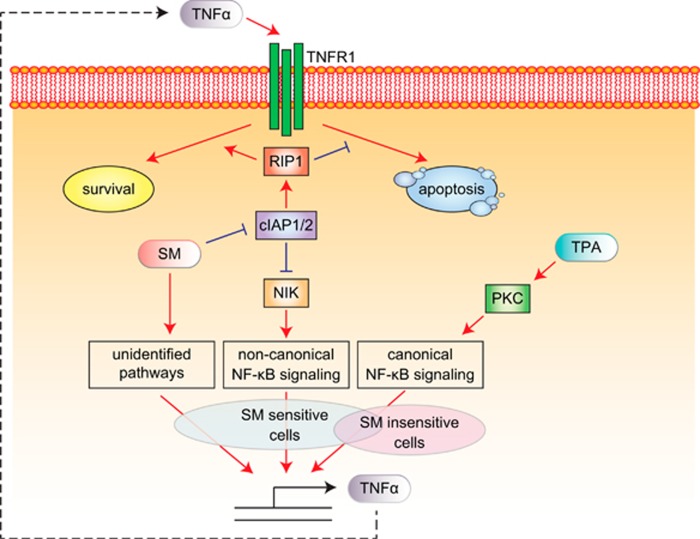
PKC activation induces TNF*α* production in cells sensitizing them to SMs. As reported by others, SM inhibits cIAP1 and cIAP2 leading to a redirection via RIP1 of the TNFR1 signaling towards apoptosis. In addition, SM-mediated cIAP1/2 inhibition leads to accumulation of NIK, propagation of the noncanonical signaling pathway and TNF*α* production in SM-sensitive cell lines. However, as the authors report this may not be the sole pathway by which SM mediates TNF*α* production in SM-sensitive cell lines, other yet unidentified pathways may also contribute to TNF*α* production. In addition, 12-O-tetradecanoylphorbol-13-acetate (TPA) that activates PKC initiates canonical NF-*κ*B signaling resulting in increased autocrine TNF*α* production and sensitizes insensitive cell lines to SM
